# Errata

**Published:** 2015

**Errata 1**

Due to a regrettable error the article **“Serotonin and the bone assessment”**, authors Carsote M, Radoi V, Geleriu A, Mihai A, Ferechide D, Opris D, Paun D, Poiana C, was published both in Journal of Medicine and Life, vol. VI, issue 2, pp. 151-155, 2013 in the “General Articles” category and in Journal of Medicine and Life Volume 7, Special Issue 2, pp. 49-53, 2014.

**The above-mentioned article will only remain valid in Journal of Medicine and Life, vol. VI, issue 2, pp. 151-155, 2013**, and the duplicate will be cancelled (will become invalid). The change should be made at all the levels: the journal’s web page – www.medandlife.ro and the databases the journal is indexed in (PubMed, Index Copernicus, EBSCO Publishing, ProQuest). 

**Errata 2**

Due to a regrettable error the article **“Detection of EWS/FLI-1 fusion in non-Ewing soft tissue tumors”**, authors Trancau IO, Huica R, Surcel M, Munteanu A, Ursaciuc C, was published both in Journal of Medicine and Life, vol. VIII, issue 1, pp. 85-89, 2015 in the “Case Presentations” category and in Journal of Medicine and Life Volume 7, Special Issue 3, pp. 114-119, 2014. 

**The above-mentioned article will only remain valid in Journal of Medicine and Life Volume 7, Special Issue 3, pp. 114-119, 2014**, and the duplicate will be cancelled (will become invalid). The change should be made at all the levels: the journal’s web page – www.medandlife.ro and the databases the journal is indexed in (PubMed, Index Copernicus, EBSCO Publishing, ProQuest).

**Errata 3**

Due to a regrettable error the article **“Biomaterials for orbital fractures repair”**,authors Totir M, Ciuluvica R, Dinu I, Careba I, Gradinaru S, was published both in Journal of Medicine and Life, vol. VIII, issue 1, pp. 41-43, 2015 in the “General Articles” category and in Journal of Medicine and Life Volume 7, Special Issue 4, pp. 62-64, 2014. 

**The above-mentioned article will only remain valid in Journal of Medicine and Life Volume 7, Special Issue 4, pp. 62-64, 2014**, and the duplicate will be cancelled (will become invalid). The change should be made at all the levels: the journal’s web page – www.medandlife.ro and the databases the journal is indexed in (PubMed, Index Copernicus, EBSCO Publishing, ProQuest). 

**Errata 4**

Due to a regrettable error the following four articles were omitted from publication in the first printed version of Journal of Medicine and Life, Volume 8, Special Issue, 2015: “**Synergistic approach to patient dialysate**”, authors Dragotoiu A, Checheriţă AI, Ciocâlteu A, Rizeanu S; “**Evaluation of adherence to anti-osteoporosis treatment from the socio-economic context**”, authors Abobului M, Berghea F, Vlad V, Balanescu A, Opris D, Bojinca V, Kosevoi Tichie A, Predeteanu D, Ionescu R; “**Socio-economical factors that influence the perception of quality of life in patients with osteoporosis**”, authors Abobului M, Berghea F, Vlad V, Balanescu A, Opris D, Bojinca V, Predeteanu D, Ionescu R and “**Study regarding the survival of patients suffering a traumatic cardiac arrest**”, authors Georgescu V, Tudorache O, Nicolau M, Strambu V, causing a modification of the numbers of the pages of the articles. 

The above-mentioned articles are now included in the final version of the journal which will not be changed anymore. 

**The publishers regret any inconvenience caused by these errors.**

